# The Associations between Biochemical and Microbiological Variables and Taste Differ in Whole Saliva and in the Film Lining the Tongue

**DOI:** 10.1155/2018/2838052

**Published:** 2018-06-14

**Authors:** Yunzi Feng, Hélène Licandro, Christophe Martin, Chantal Septier, Mouming Zhao, Eric Neyraud, Martine Morzel

**Affiliations:** ^1^School of Food Science and Engineering, South China University of Technology, Guangzhou 510640, China; ^2^Centre des Sciences du Goût et de l'Alimentation, AgroSup Dijon, CNRS, INRA, Université de Bourgogne Franche-Comté, 21000 Dijon, France; ^3^UMR A 02.102 Procédés Alimentaires et Microbiologiques (PAM), AgroSup Dijon, Université de Bourgogne Franche-Comté, 21000 Dijon, France

## Abstract

The objective of this work was to investigate whether the biological film lining the tongue may play a role in taste perception. For that purpose, the tongue film and saliva of 21 healthy subjects were characterized, focusing on microorganisms and their main metabolic substrates and products. In parallel, taste sensitivity was evaluated using a test recently developed by our group, and the links between biological and sensory data were explored by a correlative approach. Saliva and tongue film differed significantly in biochemical composition (proportions of glucose, fructose, sucrose, and lactic, butyric, and acetic acids) and in microbiological profiles: compared to saliva, tongue film was characterized by significantly lower proportions of Bacteroidetes (p<0.001) and its main genus* Prevotella* (p<0.01) and significantly higher proportions of Firmicutes (p<0.01), Actinobacteria (p<0.001), and the genus* Streptococcus* (p<0.05). Generic taste sensitivity was linked to biological variables in the two compartments, but variables that appeared influent in saliva (flow, organic acids, proportion of Actinobacteria and Firmicutes) and in tongue film (sugars and proportions of Bacteroidetes) were not the same. This study points to two interesting areas in taste research: the oral microbiome and the specific characterization of the film lining the tongue.

## 1. Introduction

Eating behavior is a key factor of health in humans: some of the major pathologies affecting the modern societies such as obesity, cardiovascular diseases, or type 2 diabetes have been reported to be strongly linked to it. Determinants of eating behavior are various and comprise, for example biological, psychological, and socioeconomic factors. Among biological factors, the sense of taste participates in the sensory perception of the food and, thus, influences food choices. Impairments in taste perception can, for example, lead to eating disorders and malnutrition [1]. Probably the most objective way of characterizing and classifying subjects according to their taste function is to measure their taste sensitivity and more precisely their individual detection threshold. This approach is thus widely used in patients with oral complaints [2, 3] or with diet-related conditions such as obesity [4, 5].

Taste perception occurs after activation of specialized receptors in the taste buds on the tongue. Saliva is the principal fluid that interacts with food and it is the medium that bathes the taste buds; thus it plays an important role in taste perception, through several mechanisms such as protection of the taste receptors or transport of taste compounds [6]. Several studies have indeed described the relationship between salivary composition and taste sensitivity, for example, to bitterness [7–9] or to the taste of oleic acid [10]. Besides free-flowing saliva, the biological film lining the tongue surface is even more intimately in contact with the taste buds. The numerous depressions of the tongue dorsum form a unique ecological site, which provides a large surface area for the accumulation of saliva, oral debris, and microorganisms [11]. Thus, the tongue is coated with a film comprising bacteria, desquamating cells, and residual saliva. The term “tongue coating” is also used in the literature although it very often refers to an undesirable excess of biological material. Some studies have linked tongue coating to taste perception. Lower overall taste performance (sensitivity to four tastants) was marginally observed in elderly patients with a coated tongue [3], and reduced recognition thresholds of saltiness and acid were observed in nursed elderly after light scraping the anterior half of the tongue [12]. Both cases support the assumption that the film lining the tongue may influence taste perception, and we formulate the hypothesis that more particularly the bacterial component of this film deserves attention. This is based on several direct or indirect observations suggesting that oral bacteria could modulate the sense of taste. Thus, Solemdal et al. [13] have made the connection between higher salivary counts of the bacteria* Streptococci* and* Lactobacilli* and reduced perception of sour taste. We also observed that a low sensitivity to the taste of fatty acids was associated with a high concentration of organic acids, probably of bacterial origin, in saliva [10]. Two possible mechanisms were mentioned: first, higher bacterial loads in the tongue film would set a barrier limiting the access of taste molecules to the taste receptors; second, bacterial metabolism may modulate the concentration of tastants (e.g., substrates such as sugars or amino-acids, end-products such as organic acids) near the taste receptors and thus modify taste sensitivity according to the sensorial adaptation mechanism.

In this context, this study had two main objectives: first, it aimed to characterize the composition of saliva and tongue film in healthy subjects, focusing on microorganisms and their main metabolic substrates and products (sugars and organics acids). Second, it aimed to investigate whether variability in these indices, especially microorganism profile, was related to variability in sensitivity to the five basic tastes (sweet, sour, salty, bitter, and umami).

## 2. Materials and Methods

### 2.1. Chemicals and Reagents

Fructose, lactose, sucrose, acetic acid, propionic acid, butyric acid, and lactic acid were obtained commercially from Sigma-Aldrich (Steinheim, Germany). Glucose was purchased from Merck (Darmstadt, Germany). The primers (Eurogentec, Belgium) used for preamplification reaction (PCR) are shown in Table 1.

### 2.2. Subjects and Sampling of Saliva and Tongue Film

The study and protocols were approved by a relevant ethical committee (Comité de Protection des Personnes Ouest V, n° 2016-A01954-47). Written informed consent was obtained from the participants. Twenty-one healthy subjects (11 females, 10 males, 22 to 60 years old) who all declared themselves to be in good oral health participated in this study. More precisely the exclusion criteria were as follows: smokers, pregnant women, food allergy sufferers, long term (over one month) medicated subjects, subjects who took an antibiotic course, had dental treatment, or used an antiseptic mouthwash in the preceding month, subjects who ever received head and neck radiotherapy, and sufferers from pathologies affecting the oral cavity (e.g., Sjögren syndrome, lichen planus, gingivitis). In addition none of the subjects brushed their tongue as part of their oral hygiene routine. Donors were instructed not to eat or drink at least 2h before sample collection, which occurred between 10 and 11 a.m. Unstimulated whole saliva was collected by direct draining into a 5 ml weighed tube during 3 minutes. After a short rest, seated participants swallowed residual saliva, immediately stuck their tongue out as far as possible, and maintained this pose while the film was taken. All samples were collected by the same experimenter. Tongue film was collected by scraping the tongue with a plastic sterile stick from the root to the apex, applying one single scraping motion. The whole sampling procedure was applied on two separate days per subject. On the first day, weights were recorded and samples were prepared for biochemical and microbiological analyses. On the second day, the pH of saliva and film samples was measured immediately after collection with a microelectrode (IQ240 pH meter, IQ Scientific Instruments, Carlsbad, CA, USA).

### 2.3. Determination of Sugars and Acids Concentration by HPLC

Saliva was diluted 1/2 and tongue film 1/4 with Milli Q water. Samples were centrifuged at 15000 g for 15 min at 4°C, and the supernatant was further diluted 1:10 and filtered through a 0.22 *μ*m nylon filter.

Sugars were analyzed using Dionex ICS-3000 ion chromatographic system (Dionex, Sunnyvale, USA) consisting of a gradient pump chromatography enclosure with a 5-*μ*L injection loop and an electrochemical detector. The sugar composition of the sample was determined by pulsed amperometric detector (PAD), using a CarboPac 1 column (2 × 250 mm i.d., 5 mm, Dionex) and 100mM NaOH as mobile phase at a flow rate of 0.25 mL/min.

Organic acids were also analyzed by Dionex ICS-3000 ion chromatography system (Dionex, Sunnyvale, USA) equipped with an electroconductivity detector. Ion chromatography was carried out using IonPac AS11-HC column (Dionex, 4×250 mm). The mobile phase, 0.8 mM NaOH, was at a flow rate of 0.25 mL/min and at room temperature. The organic acids were detected by chemical suppressed conductivity using an anion-ICE micromembrane suppressor.

Sugars and organic acids were identified and quantified according to the retention time and signal intensity of reference compounds. The standard curves were obtained using glucose, fructose, sucrose, and lactose at concentrations ranging from 0.005 to 2.5 mg/L and lactate, acetate, propionate, and butyrate at concentrations ranging from 0.15 to 37.5 mg/L.

### 2.4. Enumeration of Colony-Forming Units (CFU)

Serial dilutions (10^−3^ to 10^−6^) of diluted saliva and film samples were prepared and 100 *μ*l samples were plated on Columbia medium (Biokar) supplemented with glucose (10 g/l) and 5% (v/v) defibrinated sheep blood. Counting of colonies was performed after incubating the plates in two different conditions, aerobic and anaerobic (5% CO_2_), at 37°C for 48 h. The total concentration of cultivable microorganisms was approximated by adding the concentrations of aerobic and anaerobic microorganisms.

### 2.5. DNA Extraction and qPCR

DNA extraction was performed using Nucleo-Spin DNA tissue kit (Macherey-Nagel, Germany) following the manufacturer's instructions with a minor modification; namely, in the lysis step, 0.1 *μ*m glass beads were added and the mixture was vortexed at maximum speed for 10 min. Quality of the extracted DNA was determined using a NanoDrop ND-1000 spectrophotometer and gel electrophoresis. PCR amplifications were performed in triplicate in 20 *μ*l reaction mixtures, containing 4 *μ*l of DNA extract, 300 nM of each primer, and 10 *μ*l of SsoAdvanced™ Universal SYBR® Green Supermix (Bio-Rad) in a CFX96 Real-Time PCR Detection System (Bio-Rad, USA). Thermal cycling conditions were an initial denaturation at 95°C for 3 min followed by 40 cycles of 95°C for 15 s and 60°C for 30 s. Efficiency of amplification was determined by running a standard curve for each primers couple. Primers were selected to target total bacteria and the most representative phyla, classes, or genera of oral bacteria (Table 1). All primers targeted 16S rRNA, except for* Streptococcus* spp whose primers targeted* tuf* gene. The percentage (P) of each specific group (SPE) was determined relatively to the total bacteria (SPU) with the equation: P=(Eff. SPU)^Ctspu^/(Eff.SPE)^Ctspe^x100, with Eff being the PCR amplification efficiency. Preliminary experiments revealed that *α*-Proteobacteria was not detected in the oral microbiota. Results for the phylum Proteobacteria were therefore calculated by adding results of the two classes *β*-Proteobacteria and *γ*-Proteobacteria. Similarly, results of the genus* Fusobacteria* were considered as an approximation for the phylum Fusobacteria because it is almost exclusively the only genus of this phylum found in the oral cavity [14].

### 2.6. Determination of Taste Sensitivity Scores

**Figure 1:**
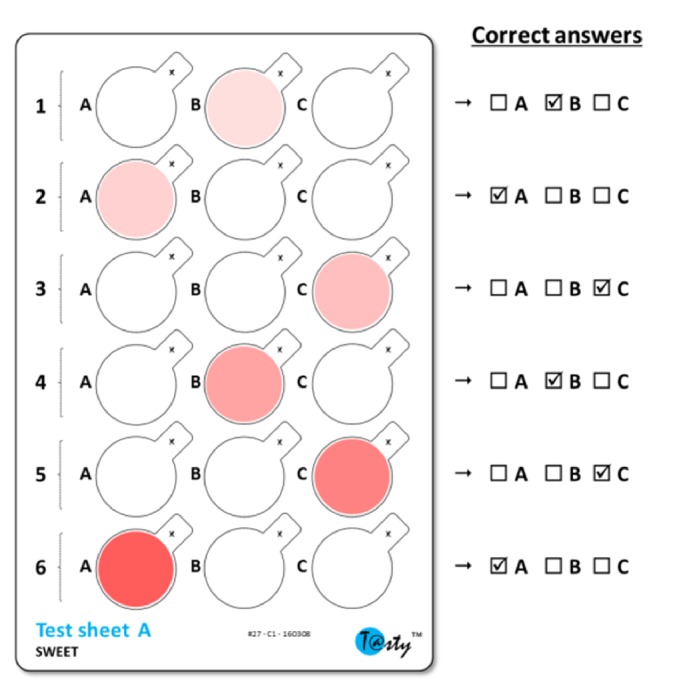
Example of a test-sheet. Tasty discs are indicated in color, with increasing color intensity corresponding to increasing concentrations of the tastant. Within each series of three discs, subjects are asked to identify which one is the tasty disc.

Taste sensitivity was evaluated for the five basic tastes: sweet (fructose), salty (sodium chloride), sour (citric acid), bitter (quinine hydrochloride), and umami (monosodium glutamate). All tastants were of food grade quality and deionized water was used as solvent. For each taste, the lowest perceived concentrations were determined thanks to a test (T@sty test™) recently developed by our group (Patent WO2015/165880). This test uses test-sheets made from edible wafer paper. A test-sheet consists of six series of three precut discs (18 mm diameter). For each series, one disc contains the tastant (the “tasty disc”) and the two others are neutral. On one test-sheet, the tastant concentration increases gradually from the first series to the sixth series (Figure 1). The concentrations were chosen to obtain a Gaussian distribution of the individual scores across the general population.

For each series, subjects were instructed to taste the three discs by placing them on the tip of the tongue for a few seconds and to find the tasty disc by answering the following question: Which disc is different from the other two? Subjects were instructed to randomly answer if no difference was perceived. Answers recorded for a full test-sheet were converted to a score ranging from 0 (low sensitivity, highest concentration not perceived) to 6 (high sensitivity, all concentrations perceived). Calculation of the score was inspired by the Best-Estimate Threshold (BET) method: each test-sheet resulted in a series of 6 answers (correct/wrong) ordered by increasing concentration of tastant. The score corresponds to the number of consecutive correct answers after the highest concentration for which a wrong answer was given. For example, a score of 2 corresponds to the case where answers given for the sixth and fifth series are correct but answer for fourth series is wrong, whatever the answers given for the other series. For each taste, the sensitivity score was the average calculated on four replicates.

### 2.7. Statistical Analysis

The statistical analysis was conducted using Statistica (StatSoft). Paired t-tests were performed to evaluate the difference in pH and composition between saliva and tongue film. Pearson correlation was calculated between pH in saliva and pH in film. The correlation between taste sensitivity scores and biological data was evaluated using Spearman correlation tests. The choice of a nonparametric test in that case is justified by the presence of censored data (scores = 0 or 6) in the sensory dataset. A score of 0 indicates that the subject would perceive the tastant only above the highest concentration presented, while a score of 6 indicates that the subject could perceive the tastant even below the lowest concentration presented.

## 3. Results

### 3.1. pH of Saliva and Tongue Film

The pH of saliva varied from 5.89 to 7.00 (mean 6.47 ± 0.29) and it was significantly lower (*p* < 0.001) than the pH of tongue film which varied from 6.53 to 7.86 (mean 7.15 ± 0.36). Moreover, a significant correlation (r = 0.839;* p* < 0.001) was observed between the pH of saliva and pH of film.

### 3.2. Salivary Flow Rate and Weight of Tongue Film

Results are depicted in Table 2. Saliva flow rates showed large variations, ranging from 0.05 to 1.13 g/min (mean 0.45 ± 0.31 g/min). The wet weights of tongue film varied between 7 and 51 mg (mean 25 ± 12 mg).

### 3.3. Sugars and Organic Acids Profiles

The concentrations and percentages of four sugars (glucose, fructose, sucrose, and lactose) and four organic acids (lactic acid, acetic acid, propionic acid, and butyric acid) in saliva and tongue film are shown in Table 3. Glucose (16.9 ± 10.5 *μ*g/g in saliva; 6.2 ± 7.0 *μ*g/g in film) and acetic acid (145.8 ± 121.5 *μ*g/g in saliva; 389.9 ± 242.3 *μ*g/g in film) were the predominant sugar and acid, respectively.

As shown in Table 3, the proportion of glucose was significantly lower (p < 0.05) in film than in saliva while the proportions of fructose and sucrose were significantly higher (p < 0.05) in film.

The proportions of lactic and butyric acid were significantly lower (*p* < 0.0001 for lactic acid,* p* < 0.01 for butyric acid) in film than in saliva while the proportion of acetic acid was significantly higher (*p* < 0.001) in film (Table 3).

### 3.4. Concentrations of Cultivable Microorganisms

**Figure 2:**
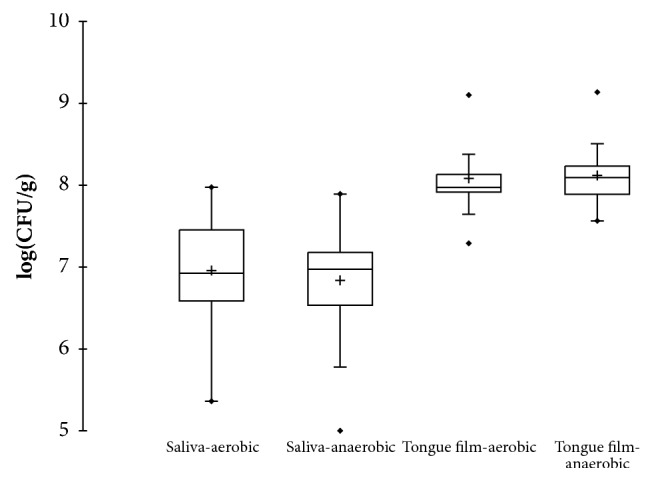
Box plot representation of cultivable aerobic and anaerobic microorganisms counts in saliva and tongue film.

The concentrations in log_10_(CFU/g) of cultivable aerobic and anaerobic microorganisms in saliva and film are shown in Figure 2. In saliva the mean concentration of aerobic microorganisms (2.10 × 10^7^ CFU/g) was slightly higher than that of anaerobic microorganisms (1.56 × 10^7^ CFU/g), whereas in film, the mean concentrations of aerobic and anaerobic microorganisms were almost similar (1.94 × 10^8^ CFU/g and 1.95 × 10^8^ CFU/g, respectively). In other words, the ratio of anaerobic/aerobic microorganisms was higher in film than that in saliva.

### 3.5. Bacterial Communities in Saliva and Tongue Film

**Figure 3:**
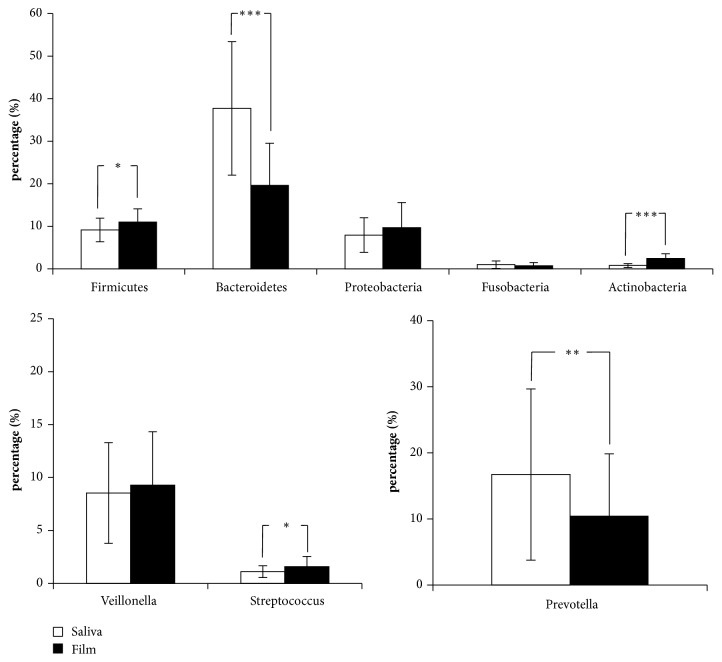
Composition of bacterial communities in saliva and tongue film (n=21): proportions of 5 phyla and 3 genera. Significant difference between saliva and tongue film is indicated as follows: *∗* p<0.05, *∗∗* p<0.01, *∗∗∗* p< 0.001.

Results for the 5 phyla and 3 genera (*Veillonella* and* Streptococcus *belonging to Firmicutes and* Prevotella* belonging to Bacteroidetes) quantified are shown in Figure 3. Overall the most abundant phyla were Bacteroidetes (37.7 ± 15.7% in saliva, 19.6 ± 9.9% in film) and Firmicutes (9.2 ± 2.8% in saliva, 11.0 ± 3.1% in film). Saliva samples were characterized by significantly higher proportions of Bacteroidetes (*p*<0.001) and its main genus* Prevotella* (*p*<0.01) whereas tongue films samples exhibited significantly higher proportions of Firmicutes (*p*<0.05), Actinobacteria (*p* <0.001), and the genus* Streptococcus* (*p* < 0.05).

### 3.6. Sensory Evaluation

**Figure 4:**
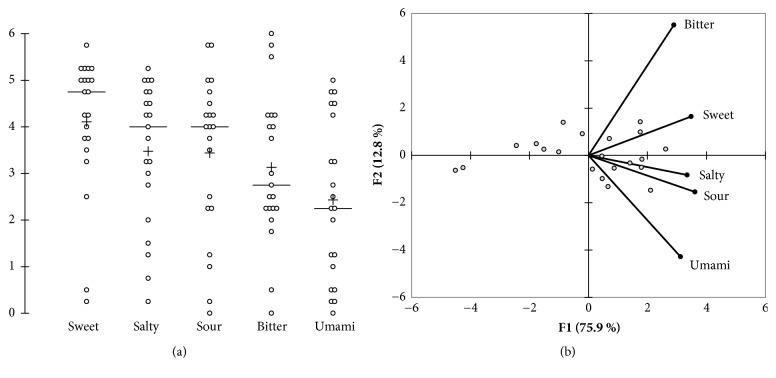
Taste sensitivity scores. Scattergram illustrating the distribution of scores across the panel (a) and biplot representation of the principal component analysis performed on the correlation matrix (b).

The mean sensitivity scores were 4.1 (sweet), 3.5 (salty), 3.4 (sour), 3.1 (bitter), and 2.4 (umami), respectively, with a wide distribution across subjects (Figure 4(a)). In addition, Figure 4(b) shows the biplot representation of the principal component analysis (PCA) performed on the correlation matrix. The first two factors represented 89% of the initial variability of the data. The F1 axis, explaining 76% of the variability, clearly differentiated subjects according to their overall sensitivity, indifferently from the taste considered. On axis 2, the main opposite contributors were scores of sensitivity to bitterness and umami. Accordingly, significant correlations (p < 0.01) were observed between sensitivity scores for all 5 tastes, at the exception of scores for bitterness and umami (r = 0.419).

### 3.7. Relationships between Taste Sensitivity Scores and Biological Variables

Table 4 shows the correlations between sensitivity scores, on the one hand, and biological data organized in 6 blocks (pH, saliva flow or film weight, concentrations of acids, concentrations of sugars, total microbial count, and proportions of the different bacterial phyla) on the other hand. To visualize the results, correlations are classified as close to null (0), weak (- or + for negative and positive correlations, respectively), or moderate (-- or ++).

Given the size of the population studied, it is not reasonable to focus on some specific correlations. However, this table provides an interesting view of some trends linking quite robustly some blocks and taste in general. In particular, saliva flow was negatively correlated to sensitivity for most tastes, and although not standing for all tastes, the negative trend was also observed for weight of the tongue film: overall, subjects with lower salivary flow and lower weights of film perceived the tastes better. There was a sharp contrast in the results linking organic acids and sensitivity scores. While the correlations were almost always positive (weak or moderate) in saliva, they were close to null or weakly negative in tongue film. Therefore, concentrations of organic acids appeared more influent in saliva than at proximity of the taste buds to explain taste sensitivity.

Looking at the sugars, there was also a clear contrast between the results in film and in saliva. This time, a lot of correlations were close to null in saliva (15 “0” out of 20 correlations), while only two “0” correlations were observed in tongue film. In tongue film, higher concentrations of glucose and fructose were systematically associated with lower sensitivity, while higher concentrations of sucrose and lactose were rather associated with higher sensitivity.

Finally, the last block where interesting and consistent correlations were found is the block of phyla proportions. In saliva, the proportions of Actinobacteria especially and Firmicutes to a slightly lesser extent were negatively associated with sensitivity scores. In tongue film, the proportion of Bacteroidetes was positively associated with three tastes.

Looking at the results from a different angle, some tastes appeared associated with more of the biological variables than others. In particular, it is interesting to note that the salty taste showed weak or moderate correlations for 14 out of the 16 variables studied in saliva, as opposed to only 7/16 for bitter and umami tastes, for example. In tongue film, the taste that showed the most and the highest correlations was bitterness.

## 4. Discussion

The objective of this work was to investigate whether the biological film lining the tongue, or tongue film, may play a role in taste perception. For that purpose, we first characterized jointly tongue film and saliva of healthy subjects and found that these two biological fluids differed in composition and microbiological profiles. Second, it was evidenced that taste sensitivity in general was linked to some biological variables in the two compartments, but variables that appeared influent in saliva (flow, organic acids, proportion of Actinobacteria and Firmicutes) and in tongue film (sugars and proportions of Bacteroidetes) were not the same.

The natural material found at the surface of the tongue is poorly described except when it is present in excess (it is then termed tongue coating), for example, in patients suffering from halitosis. The tongue film characteristics found in this study are slightly different from those of tongue coating. For example, pH of tongue coating was found to be more alkaline [15] than in the present study. The weight of film sampled here was lower than in another study [16], in which the mean weight was 173 mg (N=96). This first may be due to different sampling procedures: the tongue was scraped until no more coating could be dislodged in van Tornout et al. [16], while the tongue was scraped only once in this study. Second, subjects had different oral health status in the two studies, namely, halitosis patients versus healthy subjects. Other authors have reported an impact of oral diseases on tongue film quantity with higher wet weight of tongue film in a periodontal disease group (90.1 mg, n=17) compared to the control group (14.6 mg, n=6) [17]. The ratio of anaerobic/aerobic bacteria in tongue film here (close to 1) was also well below the value (2.29) found in tongue coating [18]. However, the authors also reported that the ratio decreased to 1.46 after treatment of halitosis by local antibiotics. Again this highlights that the available data for tongue coating do not correspond to the tongue film in a healthy situation.

In terms of biochemical composition, the concentrations found in saliva are consistent with previous reports, particularly for glucose [19] and acetate [20], while, to our knowledge, no data is available for the organic acids' composition of tongue film or tongue coating. The origin of the organic acids in the oral cavity is repeatedly attributed to microbial metabolism. This has been described particularly for the production of lactic acid from glucose or sucrose in saliva and in dental plaque [21, 22] where the large increase (5 to 8-fold change) in lactic acid concentration is both rapid (within 5 minutes) and transient (returning to basal level in approximately 30 minutes). However, when the extracellular sugar supply is limited, for example, some time away after a meal like in our conditions, bacterial metabolism is not dominated by lactate production but shifts to production of mixed acids [23]. Thus both in saliva [22] and in dental plaque [21] the predominant organic acids in resting conditions are ordered as in the present study, i.e., acetic acid > propionic acid > lactic acid > butyric acid. Our results indicate that the tongue film of healthy individuals is comparatively richer in acetic acid than saliva, which may be linked to different bacterial communities. For example,* Veillonella* utilizes lactate specifically and produces acetate and propionate as end-products. Other Firmicutes such as* Lactobacilli* and* Streptococci* can also convert lactate to acetate, as also* Actinomyces* belonging to the Actinobacteria phylum [23]. In our case, we indeed found that the two phyla Firmicutes and Actinobacteria were more represented in tongue film.

The 5 phyla quantified account for 80-95% of the entire saliva microbiome [24] and are also abundant in other oral samples [25, 26]. Our results differ from other reports in which Firmicutes was often the most abundant phylum in saliva [27], in tongue coating [28] or on the tongue dorsum [26]. This divergence is likely linked to different DNA extraction methods, but it does not prevent comparison between subjects or correlations with taste sensitivity scores.

Focusing now on the sensory results, the correlative approach we chose in this study has been seldom applied. Most articles linking saliva properties and composition and taste sensitivity have opted for an approach where groups were constituted based on sensory results and differences between groups were tested [7–10]. However, one recent study correlated salivary biochemical data and taste sensitivity [29]. Their main finding was that sweetness sensitivity correlated with salivary pH but only for 38% of subjects with the highest and lowest pH values. Keeping all subjects in our study, the link between pH and sensitivity is not clearly established neither in saliva nor in tongue film.

We found consistent negative correlations between taste sensitivity and saliva flow/tongue film weight. Concerning saliva, comparable correlations between flow and taste sensitivity have been previously described. For example, when an acid enters the oral cavity, a high salivary flow (usually associated with higher buffering capacity of saliva) diminishes the protons concentration at the receptors vicinity [6] and therefore sensitivity would be lowered. Similar associations were reported between salivary flow and sensitivity to oleic acid [30] or NaCl perception [6]. It is worth mentioning that methods used to determine taste sensitivity are most often performed with tastants in solution, which implies that the taste solution is diluted in saliva present in the oral cavity. In our case, the tastant is included in a solid matrix placed on the tongue, but the correlation also indicates that a low salivary flow improves sensitivity. It is therefore highly plausible that the amount of saliva on the tongue is related to saliva flow and that at low flow (yet within the healthy range), the local tastant concentration is higher. In addition, the tongue film weight was also negatively correlated to sensitivity, translating that the film also acts as a barrier limiting the diffusion of the taste molecules to the receptors on the tongue. This is, for example, consistent with Quirynen et al. [31] who found a lowering of taste identification threshold after mechanical cleansing of tongue treatment, resulting in a lower index of tongue coating. The removal of tongue coating by mild brushing has also been previously shown to improve sour taste recognition in older adults [12].

The correlation between taste sensitivity and organic acids concentrations were more numerous and stronger in saliva than in tongue film and clearly indicate that higher salivary levels of organic acids are associated with a higher sensitivity. This relationship is intriguing and opposite to a previous finding where higher organic acids concentrations in saliva were associated with lower sensitivity to the taste of fat [10]. To date, the mechanism linking organic acids and taste sensitivity remains unknown. In contrast to organic acids, the correlation between taste sensitivity and sugars were more numerous and stronger in tongue film than in saliva. First it should be noted that, contrarily to all the organic acids which may be produced within the oral cavity, three of the four sugars measured are brought to the oral cavity through food intake (fructose found in fruit, lactose found in milk, and sucrose produced from plant sources). It therefore seemed initially plausible that the variations in levels of these sugars on the tongue film corresponded to the capacity of naturally cleansing the tongue after food intake. However in the present study, the signs of correlations were different between taste and fructose, on the one hand, and taste and lactose and sucrose, on the other hand. This suggests that the correlations do not merely translate a generic capacity of tongue cleansing. Another explanation may therefore reside in sugar metabolism found on the tongue dorsum. The two sugars which tended to be positively associated with taste sensitivity were the disaccharides sucrose and lactose, while the monosaccharides fructose and glucose were negatively associated with taste sensitivity. In mammalians, the disaccharides can be hydrolyzed by intestinal enzymes, and most oral bacteria can also use disaccharides for glycolysis. Perhaps more interestingly, sucrose specifically can be converted by oral bacteria into glucan and fructan, which serve as “building material” for biofilms [23]. This phenomenon has been mainly described for* Streptococci* in the context of bacterial adhesion to the dental surfaces [32], but glycoproteins have also been observed surrounding bacteria adherent to mucosal cells of tongue rats [33]. If such glycoproteins are at least partly of bacterial origin, this would mean that the sucrose conversion to glucan/fructan participates in the strength of biofilms on the tongue surface. Therefore, a higher level of sucrose in the tongue film may indicate a lower proportion of bacteria capable of converting them into glucan and fructan (or a lower conversion rate) and therefore a less firmly structured biofilm. The physical barrier between tastants and taste receptors would, as a consequence, be less efficient and sensitivity increased. A finer characterization of the bacterial genera and species in the tongue film and targeted study of their sugar metabolisms would be necessary to test this hypothesis. This conclusion also stands when looking at the bacteriological results: at present, it is suggested that higher proportions of Actinobacteria and Firmicutes in saliva are linked to lower taste sensitivity, while a higher proportion of Bacteroidetes in the tongue film increases sensitivity (particularly to bitterness). Given the diversity of genera and species within a phylum, described elsewhere in detail for the oral microbiome [34], it is overall difficult to propose mechanistic explanations on those links. This is especially true for the oral cavity, where particularly high within-subject diversity was reported [26]. Again, a more detailed characterization of microbial communities and of their metabolism would be of interest, but this study demonstrates that the oral microbiome deserves to be considered as an explanatory variable when investigating perireceptor events involved in taste perception. To conclude, one should keep in mind that only a few correlations between taste sensitivity scores and biological variables were significant, and such correlations were modest. In addition, this preliminary study was conducted on a limited number of subjects and should be extended to a larger panel. Nevertheless, in spite of such limitations, this work already points to two interesting areas in taste research: the oral microbiome in general and the specific characterization of the film lining the tongue.

## Figures and Tables

**Table 1:** Primers used for qPCR assays.

**Primer **	**Oligonucleotide sequence (**5′-3′**)**	**Target**	**Reference**
SPU Fwd	AAACTCAAAKGAATTGACGG	All bacteria	a
SPU Rev	CTCACRRCACGAGCTGAC	All bacteria	a
ACT Fwd	TACGGCCGCAAGGCTA	Actinobacteria	a
ACT Rev	TCRTCCCCACCTTCCTCCG	Actinobacteria	a
BACT Fwd	CRAACAGGATTAGATACCCT	Bacteroidetes	a
BACT Rev	GGTAAGGTTCCTCGCGTAT	Bacteroidetes	a
FIRM Fwd	TGAAACTYAAAGGAATTGACG	Firmicutes	a
FIRM Rev	ACCATGCACCACCTGTC	Firmicutes	a
*γ*-P Fwd	TCGTCAGCTCGTGTYGTGA	*γ*-Proteobacteria	a
*γ*-P Rev	CGTAAGGGCCATGATG	*γ*-Proteobacteria	a
*β*-P Fwd	ACTCCTACGGGAGGCAGCAG	*β*-Proteobacteria	b
*β*-P Rev	TCACTGCTACACGYG	*β*-Proteobacteria	b
Fuso Fwd	CGCAGAAGGTGAAAGTCCTGTAT	*Fusobacterium* spp	c
Fuso Rev	TGGTCCTCACTGATTCACACAGA	*Fusobacterium *spp	c
Veil Fwd	A(C/T)CAACCTGCCCTTCAGA	*Veillonella* spp	d
Veil Rev	CGTCCCGATTAACAGAGCTT	*Veillonella* spp	d
Strep Fwd	GTACAGTTGCTTCAGGACGTATC	*Streptococcus* spp	e
Strep Rev	ACGTTCGATTTCATCACGTTG	*Streptococcus* spp	e
Prev Fwd	CACCAAGGCGACGATCA	*Prevotella* spp	f
Prev Rev	GGATAACGCCYGGACCT	*Prevotella* spp	f

^a^ De Gregoris et al., 2011; ^b^ Pécastaings et al., 2016; ^c^ Suzuki et al., 2004; ^d^ Rinttilä et al., 2004; ^e^ Picard et al., 2004; ^f^ Marathe et al., 2012.

**Table 2:** Salivary flow rates and weight of tongue film sampled (n=21).

	**Mean**	**Min**	**Max**	**Median**	**Standard **	**SD **%
					**deviation**	
					** (SD)**	
Salivary flow rates	0.45	0.05	1.13	0.36	0.31	68.6
(g/min)						
Weight of tongue film	0.025	0.007	0.051	0.020	0.012	48.5
(g)						

**Table 3:** Concentrations and relative proportions (percentages) of sugars and organic acids in saliva and tongue film. The p value represents the level of significance when comparing the relative proportions of each metabolite between saliva and film (paired t-test).

**Compound**	**Concentration in**	**Concentration in**	**Proportion in **	**Proportion in **	**p**
	** saliva (** **µ** **g/g**±**SD)**	** film (** **µ** **g/g**±**SD)**	**saliva (**%±** SD)**	**film (**%±** SD)**	
glucose	16.9 ± 10.5	6.2 ± 7.0	95.4 ± 10.5	89.7 ± 7.0	<0.05
fructose	0.3 ± 0.3	0.3 ± 0.2	1.6 ± 0.3	3.6 ± 0.2	<0.05
sucrose	0.1 ± 0.1	0.2 ± 0.3	0.4 ± 0.1	2.4 ± 0.3	<0.05
lactose	0.5 ± 0.3	0.3 ± 0.5	2.7 ± 0.3	4.3 ± 0.5	ns
lactate	15.0 ± 19.1	5.1 ± 3.1	10.9 ± 11.5	1.3 ± 0.9	<0.001
acetate	145.8 ± 121.5	389.9 ± 242.3	67.6 ± 9.4	80.1 ± 7.1	<0.0001
propionate	44.9 ± 55.8	96.5 ± 96.9	16.2 ± 6.3	16.2 ± 6.3	ns
butyrate	12.6 ± 14.1	12.0 ± 15.1	5.4 ± 3.9	2.3 ± 2.9	<0.01

**Table 4:** Correlations between taste sensitivity scores and biological variables in saliva and in tongue film. The Spearman correlations coefficients are coded as follows: -- for -0.5< r < -0.3 (moderate negative correlation); - for -0.3< r < -0.1 (weak negative correlation); 0 for -0.1<r<0.1; + for 0.1< r<0.3 (weak positive correlation); ++ for 0.3< r < 0.5 (moderate positive correlation). A star indicates a significant correlation (p<0.05).

**Salivary variables**	**Sweet**	**Salty**	**Sour**	**Bitter**	**Umami**	**Film variables**	**Sweet**	**Salty**	**Sour**	**Bitter**	**Umami**
pH	0	+	-	+	0	pH	0	0	0	0	0

flow	-	--	--	0	--*∗*	weight	-	0	-	0	--

lactate	++	+	+	+	+	lactate	-	-	0	-	0
acetate	+	++	+	+	0	acetate	-	0	0	-	0
propionate	+	++*∗*	+	+	+	propionate	-	0	-	0	0
butyrate	++	++	+	+	0	butyrate	0	0	0	0	-

glucose	0	0	0	0	0	glucose	-	-	-	--	-
fructose	0	++	+	0	+	fructose	-	-	-	--	--
sucrose	-	-	-	0	-	sucrose	++	0	+	+	+
lactose	0	0	0	0	0	lactose	+	0	+	-	+

total bacterial count	0	+	0	0	0	total bacterial count	-	+	-	0	0

Actinobacteria	--	--*∗*	-	-	--	Actinobacteria	0	0	-	+	-
Bacteroidetes	0	+	-	0	-	Bacteroidetes	+	+	0	++*∗*	0
Firmicutes	--	-	-	-	0	Firmicutes	0	0	0	-	-
Proteobacteria	0	-	0	0	+	Proteobacteria	0	-	0	0	+
Fusobacteria	0	-	0	0	0	Fusobacteria	0	-	-	0	0

## Data Availability

The datasets generated and/or analyzed during the current study are available from the corresponding author on reasonable request.
